# Gender stereotypes and the construction of self-beliefs in spatial cognition

**DOI:** 10.1007/s00426-026-02303-8

**Published:** 2026-05-19

**Authors:** Linda Arrighi, Markus Hausmann

**Affiliations:** Department of Psychology, Durham University, Durham, UK

## Abstract

Specific spatial abilities such as the Mental Rotations Test have consistently shown pronounced sex/gender differences. Previous research has investigated biological (e.g., hormones), psychological (e.g., self-beliefs about abilities), and social factors (e.g., sex/gender stereotypes) to explain these sex/gender differences. However, the combined effects of two or more of these factors have rarely been investigated. Here, we explored the complex interaction between sex/gender stereotypes and self-beliefs in spatial abilities, as well as their effects on sex/gender differences in mental rotation performance. Participants (*n* = 130; 50 males, 80 females) rated their self-beliefs in spatial abilities and subsequently completed two sex-/gender-sensitive paper-pencil mental rotation tasks that varied in task demands (adapted from Jäger & Althoff, 1994 and Peters 1995), following either sex/gender or neutral priming through a priming questionnaire. The results indicate that, while sex/gender differences in self-beliefs and mental rotation performance were significant, sex/gender priming did not directly influence males’ or females’ self-beliefs or mental rotation performance. Nevertheless, self-beliefs and the endorsement of sex/gender stereotypes consistently predicted females’ mental rotation performance. These findings suggest that sex/gender differences in (spatial) cognitive performance—specifically, men outperforming women—primarily emerge when women believe less in their abilities and hold stronger sex/gender stereotypes. Future research should routinely assess individuals’ beliefs in their cognitive abilities and endorsement of sex/gender stereotypes when investigating cognitive group differences (e.g., cognitive sex/gender differences), to avoid overlooking their potential role in shaping cognitive performance.

##  Introduction

Men's and women’s cognitive performance largely overlaps in many cognitive domains (Halpern & LaMay, 2000; Hyde, 2016). Nevertheless, men tend to excel in specific spatial tasks (e.g., Reilly & Neumann, 2013; Voyer et al., 1995), whereas women tend to excel in specific verbal (e.g., Hirnstein et al., 2023) and memory tasks (e.g., Asperholm et al., 2019; Voyer et al., 2007). Regarding sex/gender^1^ differences in spatial cognition, previous research has revealed small to moderate effect sizes in spatial visualisation (*d* = 0.13–0.19), visuospatial working memory (*d* = 0.20), navigation (*d* = 0.34), and spatial perception (*d* = 0.44; Linn & Petersen, 1985; Nazareth et al., 2019; Voyer et al., 1995, 2017).

Another spatial task known to be sex-/gender-sensitive is mental rotation, defined as the ability to visualise and mentally rotate abstract figures (Shepard & Metzler, 1971). Certain versions of the task, such as the Revised Vandenberg & Kuse Mental Rotations Tests (MRT; Peters et al., 1995), have shown the largest cognitive sex/gender difference (*d* = 0.56–0.73) within psychological literature (Zell et al., 2015).

The present study aimed to advance our understanding of the origins of sex/gender differences in (spatial) cognitive performance by employing (1) two mental rotation tasks as models for sex-/gender-sensitive cognitive tasks and (2) a psychosocial approach to investigate potential interactions between psychological (i.e., self-perception of spatial ability) and sociocultural (i.e., sex/gender stereotypes) factors. Many researchers have argued that cognitive sex/gender differences are unlikely to arise from a single source and are best understood through a biopsychosocial lens (e.g., Halpern & LaMay, 2000; Hausmann, 2021).

In addition to psychosocial factors, biological factors such as sex hormones levels have been investigated in this context, yielding mixed results (e.g., Hausmann et al., 2009; Hromatko & Tadinac, 2024; Sanchis-Segura et al., 2018). The present study, however, focused exclusively on psychosocial factors, based on the hypothesis that the origin of sex/gender differences in (self-perceived) spatial ability may relate to the endorsement of pronounced sex/gender stereotypes about spatial cognition (Lourenco & Liu, 2023). This suggests the importance of examining the interplay between psychological and sociocultural factors in more depth.

### Self-perception of spatial abilities

Individuals’ self-perception of their spatial abilities is a key contributor to (spatial) cognitive sex/gender differences. Although often overlooked, such self-beliefs have been shown to predict mental rotation performance (Alvarez-Vargas et al., 2020; Arrighi & Hausmann, 2022; Cooke-Simpson & Voyer, 2007; Estes & Felker, 2012). Self-perception of spatial abilities consistently emerged as a sex-/gender-sensitive construct, with men generally holding more positive self-evaluations of their spatial abilities than women (e.g., Lyons et al., 2018; Matthews et al., 2024), even in contexts where objective performance differences were absent (Ariel et al., 2018; Picucci et al., 2011; Reilly et al., 2022). A recent study found that women’s lower self-reported spatial intelligence was driven by underestimation of their own abilities, whereas men’s self-assessments tended to more accurately reflect performance (Hofer et al., 2025). Given the sex-/gender-sensitive nature of these self-perceptions, it is important to consider whether they directly contribute to cognitive sex/gender differences.

Regarding the origins of these self-perceptions, it has been proposed that the sociocultural environment, particularly sex/gender stereotypes about cognition, shapes individuals’ beliefs about their own abilities, thereby influencing cognitive performance (Lourenco & Liu, 2023). Moreover, the relationship between sex/gender stereotypes, self-perception, and cognitive performance may be bidirectional. That is, self-perception of one’s own abilities may also affect the degree to which individuals internalise or resist sex/gender stereotypes, with consequences on cognitive performance. As this interaction has not yet been empirically tested, the present study investigated (1) whether sex/gender stereotypes priming influences self-perception and (spatial) cognitive performance, and (2) whether the effect of such priming on cognitive performance is moderated by self-perception.

The present study focused on three sex/gender-sensitive self-perception variables related to spatial abilities: spatial anxiety (i.e., apprehension around spatial tasks; Lawton, 1994; Ramirez et al., 2012), self-efficacy in spatial ability (i.e., confidence in one’s ability to successfully complete spatial tasks; Arrighi & Hausmann, 2022; Towle et al., 2005), and self-confidence (i.e., momentary self-assessment of spatial task performance; Cooke-Simpson & Voyer, 2007; Estes & Felker, 2012). Although these three constructs overlap, particularly spatial anxiety and self-efficacy, which are often significantly correlated (Arrighi & Hausmann, 2022; Kaufmann et al., 2022; Razavi et al., 2017), they differ conceptually. Spatial anxiety and self-efficacy reflect relatively stable (Muffato et al., 2023), trait-like aspects of spatial self-concept, whereas self-confidence represents a more transient, item- and task-specific self-assessment. The inclusion of both trait and state self-perception variables within the current study allowed to investigate whether the effects of and interactions with sex/gender priming were temporary (only acting on state variables) or potentially longer-term (also acting on more stable trait variables).

Several studies have shown that self-perception of spatial abilities, including the three variables investigated here, predicts mental rotation performance in both men and women (Alvarez-Vargas et al., 2020; Arrighi & Hausmann, 2022; Cooke-Simpson & Voyer, 2007; Estes & Felker, 2012; Fioriti et al., 2024). However, some recent evidence challenges this relationship (Desme et al., 2024), suggesting the need for further research to elucidate the mechanisms and directionality between self-perception and cognitive performance (Fioriti et al., 2024). Understanding these mechanisms may, in turn, clarify the origins of (spatial) cognitive sex/gender differences.

### Sociocultural factors

Sex/gender stereotypes about cognitive abilities (e.g., *“men have better spatial abilities than women”*) are widely held in the general population (Halpern et al., 2011; Halpern & Tan, 2001) and represent one of the most powerful sociocultural influences on performance in sex-/gender-sensitive tasks such as mental rotation (Miller & Halpern, 2014).

Empirical studies have used sex/gender priming to investigate how such stereotypes affect cognitive performance. Depending on the specific stereotype activated, performance may improve or decline. For example, activating a positive stereotype about one’s in-group can enhance performance (*stereotype boost*; Shih et al., 2002). Activating a negative stereotype about an out-group can have a similar effect (*stereotype lift*; Walton & Cohen, 2003). In contrast, activating a negative stereotype about one’s in-group can result in performance declines (*stereotype threat;* Steele, 1997), although some individuals may be motivated to increase effort and thus performance to disprove the stereotype (*stereotype reactance;* Kray et al., 2001).

Sex/gender priming can occur through explicit cues (e.g., informing participants of stereotypes before a task; Nguyen & Ryan, 2008; Wulandari & Hendrawan, 2021) or implicit cues (e.g., asking participants to report their sex and/or gender before a task; Nguyen & Ryan, 2008; Wulandari & Hendrawan, 2021). Even subtle implicit primes can affect performance (Drążkowski et al., 2017; Martens et al., 2006; McGlone & Aronson, 2006; Nguyen & Ryan, 2008; Ortner & Sieverding, 2008; Sharps et al., 1994). Critically, many studies not explicitly focused on sex/gender differences may have unintentionally primed participants, potentially contributing to inconsistencies across the literature.

Studies investigating the effects of sex/gender stereotypes on mental rotation performance have found that sex/gender primed women typically perform worse than non-primed women, consistent with *stereotype threat* (e.g., Hausmann et al., 2009; Hirnstein et al., 2014; Moè, 2009; Sanchis-Segura et al., 2018). On the other hand, sex/gender primed men often perform better than non-primed men, consistent with *stereotype boost* (e.g., Guizzo et al., 2019). Even when primed with negative stereotypes about their in-group, men tend to perform comparably better in language-related tasks (e.g., Chaffee et al., 2020; Hirnstein et al., 2012), suggesting that women are generally more susceptible to *stereotype threat*. Moreover, effect sizes of sex/gender differences are often larger in primed than in non-primed samples (e.g., Hausmann et al., 2009; Hirnstein et al., 2014; Moè, 2009; Sanchis-Segura et al., 2018). Nonetheless, it remains unclear whether sex/gender stereotypes directly contribute to cognitive sex/gender differences. Meta-analyses indicate that *stereotype threat* effects on women’s cognitive performance are generally small (e.g., *d* = 0.26; Nguyen & Ryan, 2008) and difficult to replicate (Flore & Wicherts, 2015; Nguyen & Ryan, 2008; Stoet & Geary, 2012), suggesting substantial confounding factors.

Some studies have explored conditions under which the effects of sex/gender priming are amplified or attenuated, focusing on sample and task characteristics. For example, women with a science background who were sex/gender primed performed better than non-primed counterparts, suggesting *stereotype reactance* (Hausmann, 2014), possibly related to their dual identification as both women and scientists (Cromley et al., 2013; Hausmann, 2014; McGlone & Aronson, 2006; Sanchis-Segura et al., 2018).

Susceptibility to priming also depends on participants endorsement of sex/gender stereotypes (Hausmann et al., 2009; Moè et al., 2021). For women, stronger endorsement of such stereotypes correlated with lower mental rotation performance (Hausmann et al., 2009), suggesting that *stereotype threat* effects may primarily occur among those holding pronounced stereotypes. Task characteristics can further modulate these effects, with *stereotype threat* being more pronounced when task demands are high (e.g., Allison et al., 2017; Keller, 2007).

In sum, existing evidence supports the notion that sex/gender stereotypes can substantially influence men’s and women’s (spatial) cognitive performance. However, it remains uncertain whether they directly contribute to cognitive sex/gender differences. Moreover, their effects are often shaped by sample characteristics, task demands, and potentially by individuals’ self-perception of spatial abilities—a moderating factor examined in the present study.

### Psychosocial approach

The findings reviewed above suggest that both psychological and sociocultural factors contribute to (spatial) cognitive sex/gender differences. However, only a limited number of studies have investigated multiple such factors simultaneously (e.g., Halpern & LaMay, 2000; Hausmann, 2021).

Supporting the idea that sex/gender stereotypes and self-perception of spatial ability intersect, Rahe et al. (2023) demonstrated that individuals’ self-perception of their own abilities can influence their susceptibility to sex/gender stereotypes, with downstream effects on cognitive performance. Specifically, Rahe et al. (2023) showed that adolescents’ stronger self-concept was associated with higher mental rotation scores among sex/gender primed boys and lower scores in non-primed boys. Conversely, among girls, a stronger self-concept predicted lower mental rotation scores in the sex/gender primed condition and higher scores in the non-primed condition. These results suggest that the moderating effect of self-perception on the influence of sex/gender priming on performance is sex-/gender-specific. What remains unclear, however, is whether susceptibility to sex/gender priming is influenced by self-perception. Specifically, it is unknown whether self-perception variables directly moderate the effect of sex/gender priming on cognitive performance—i.e., whether individuals with lower self-perceived (spatial) abilities are more likely to experience *stereotype threat* than those with higher self-perceived abilities following explicit priming (e.g., being told that men outperform women on the upcoming task, Moè, 2009) or implicit priming (e.g., emphasising the spatial nature of the task, Sharps et al., 1994).

To address this question, the present study employed a theoretical framework informed by the *biopsychosocial model of challenge and threat* (Blascovich, 2008; Blascovich & Mendes, 2000). Grounded in cognitive appraisal theory (Lazarus & Folkman, 1984), this theoretical model sets out to explain how, in stressful performance situations, people’s subjective evaluation of the demands of the task and their own resources determines emotional and behavioural responses, and specifically whether they experience a physiological challenge state (adaptive) or a physiological threat state (maladaptive; Blascovich & Mendes, 2000). When individuals appraise a task as within their capabilities, they experience a challenge state, accompanied by increased cardiac output, efficient blood flow, and generally better (cognitive) performance; when they perceive it as exceeding their abilities, they experience a threat state, accompanied by higher vascular resistance, increase in stress hormones levels, and generally worse (cognitive) performance (Blascovich, 2008).

This framework offers a comprehensive lens through which to interpret why sex/gender priming manipulations may elicit diverse and sometimes opposing effects across individuals and tasks (e.g., stereotype *boost/lift*, *threat*, or *reactance*). Applied to sex/gender priming, the model suggests that individuals with more positive self-perception of their (spatial) cognitive abilities—particularly men—may appraise the priming situation as challenging, leading to enhanced mental rotation performance. Conversely, those with lower self-perception—particularly women—may experience the priming situation as threatening, resulting in lowered performance.

Supporting this interpretation, Hausmann et al. (2009) found that increases in mental rotation performance among sex/gender primed men (i.e., *stereotype boost*) were accompanied by elevated testosterone levels compared to non-primed men, consistent with a challenge-related physiological response. In contrast, *stereotype threat* effects observed in women may reflect a threat-related appraisal. Indeed, previous studies have shown that sex/gender priming can influence physiological stress markers, including heart rate and cardiac output (Vick et al., 2008), as well as increased activation in brain areas associated with emotional processing (i.e., left rostral–ventral anterior cingulate and right orbital gyrus; Wraga et al., 2006). These findings suggest that sex/gender priming can induce high stress or threatening states (Herd, 1991).

Taken together, the results imply that *stereotype boost* effects are likely associated with challenge appraisals, whereas *stereotype threat* effects are linked to threat appraisals. However, it remains unclear whether susceptibility to either outcome is also related to individuals’ self-perception of their (spatial) abilities. The present study therefore sought to enhance understanding of the underlying mechanisms of *stereotype boost* and *threat*, focusing on self-perception variables as potential moderators.

### Study aims and hypotheses

The present study investigated the complex interaction between individuals’ self-perception of (spatial) abilities and sex/gender stereotypes, and how this interaction may influence sex/gender differences in a sex-/gender-sensitive cognitive task (e.g., MRT). We hypothesised that males outperform females in mental rotation and report greater self-confidence in their mental rotation abilities, particularly following sex/gender priming (Hypothesis 1). We further predicted that males report lower spatial anxiety, higher self-efficacy, and higher self-confidence in mental rotation than females overall (Hypothesis 2). We expected baseline levels of spatial anxiety and self-efficacy to moderate the effects of sex/gender priming in both males and females. Specifically, we hypothesised *stereotype boost/reactance* effects (i.e., improved performance) among participants with lower spatial anxiety and higher self-efficacy, and *stereotype threat* effects (i.e., lowered performance) among participants with higher spatial anxiety and lower self-efficacy (Hypothesis 3).

Finally, a series of exploratory analyses examined whether participants in the experimental condition endorsed sex/gender stereotypes. Additional exploratory regression analyses investigated whether self-perception and sex/gender stereotypes regarding spatial abilities predicted mental rotation performance in a sex-/gender-specific manner.

All hypotheses were pre-registered prior data collection on AsPredicted.org (https://aspredicted.org/f5tp-wsz3.pdf).

## Methods

### Participants and design

A total of 144 participants (51 males and 93 females, based on birth-assigned sex) were recruited for the present study. Of these, 61 participants were recruited from the Department’s student participation pool and 83 from advertisements across the University. As incentives, participants could enter a prize draw for five vouchers (£10–20 each). In addition, psychology students at Durham University received course credits for participation.

The study was advertised as investigating the relationship between stress and cognition, with no mention of sex/gender or the specific task domain (i.e., spatial cognition). Pre-screening excluded individuals who were undergoing hormone therapy, using medication affecting the central nervous system and/or the HPA axis (excluding hormonal contraceptives), or reporting neurological, psychiatric, endocrine, cardiovascular, or other chronic conditions. Participants had normal or corrected-to-normal vision and hearing. Participants were naïve to the study hypotheses and its focus on sex/gender.

This study was approved by the Departmental Ethics Committee. Written informed consent was obtained from all participants, and their privacy rights were fully protected.

Fourteen participants were excluded from the final analyses due to (1) incomplete data (*n* = 2), (2) extremely poor MRT performance (score of zero, *n* = 1), or (3) prior participation in similar studies by the same research group, resulting in familiarity with the materials or hypotheses (*n* = 11). The final sample therefore comprised 130 participants (50 males, 80 females).

Males (21.46 ± 4.31 years, M ± SD; range: 18–40 years) and females (21.10 ± 3.97 years; range: 18–37 years) were similar in age, *t*_(128)_ = 0.49, *p* =.627, *d* = 0.09. Because grouping was based on birth-assigned sex, the terms *male* and *female* are used. However, given that most participants identified as cisgender, and the small number of gender diverse participants precluded separate analyses, the combined term *sex/gender* is used throughout. Detailed demographic characteristics are presented in Table 1.

**Table 1:** Detailed demographic information listed as absolute frequencies (and percentages)

Characteristics	Males	Females
***N***	50	80
**Gender identity**		
Man	46 (92)	1 (1.2)
Woman	0 (0)	77 (96.3)
Nonbinary	0 (0)	2 (2.5)
Prefer not to say/Not sure	4 (8)	0 (0)
**Field of study/career**		
Psychology	7 (14)	42 (52.5)
Science, technology, engineering, and mathematics (STEM; incl. biology)	25 (50)	11 (13.75)
Applied and social sciences	7 (14)	13 (16.25)
Humanities	11 (22)	14 (17.5)
**Education**		
GCSE	1 (2)	0 (0)
High School/College	37 (74)	62 (77.5)
Bachelor’s	7 (14)	10 (12.5)
Master’s/PGCert	4 (8)	7 (8.75)
PhD	1 (2)	1 (1.25)
**Ethnicity**		
White	35 (70)	42 (52.5)
Asian	7 (14)	28 (35)
Black	3 (6)	1 (1.25)
Mixed/Other/Missing	5 (10)	9 (11.25)
**Sexual orientation**		
Heterosexual	38 (76)	53 (66.25)
Bisexual/Pansexual	4 (8)	15 (18.75)
Gay/Lesbian	2 (4)	6 (7.5)
Asexual	2 (4)	0 (0)
Prefer not to say/Other/Missing	4 (8)	6 (7.5)
**Days since start of cycle**		
1–5 days	N/A	22 (27.5)
6–10 days	N/A	11 (13.7)
10–14 days	N/A	10 (12.5)
14–20 days	N/A	9 (11.3)
20–24 days	N/A	7 (8.7)
24–28 days	N/A	5 (6.3)
28 + days	N/A	13 (16.25)
Not having periods/Unsure	N/A	3 (3.75)
**Contraceptive use**		
None	N/A	62 (77.5)
Hormonal contraceptives	N/A	14 (17.5)
Copper IUD	N/A	3 (3.8)
Prefer not to say/Missing	N/A	1 (1.2)
**Previous mental rotation experience**		
Naïve to mental rotation tasks	23 (46)	36 (45)
Not naïve to mental rotation tasks	27 (54)	37 (46.2)
No answer	0 (0)	7 (8.8)

The study applied a 2 (sex/gender: male, female) x 2 (priming condition: sex/gender primed, neutral control) between-participants design. A priori power analysis (G*Power v3, α = 0.05, power = 0.80, effect size *f* = 0.25 based on similar previous literature investigating the interaction of sex/gender and stereotype priming, e.g., Hausmann et al., 2009; Moè, 2009; Sanchis-Segura et al., 2018) indicated a minimum required sample size of *n* = 128 for detecting fixed, main, and interaction effects in an ANOVA (groups = 4, numerator df = 1).

### Materials

#### Self-efficacy in spatial abilities

Participants rated their confidence in successfully completing 12 spatial problems on a 7-point Likert scale (1 = *not at all confident in my ability*, 7 = *highly confident in my ability*). Means were used for analysis (range: 1–7). Cronbach’s α for the normative (Hausmann, 2026) and the present sample were 0.93, and 0.87, respectively.

#### Spatial anxiety

Spatial anxiety was assessed using 12 items adopted from Arrighi and Hausmann (2022). Participants rated the extent they identified with statements describing feelings and experiences related to spatial activities on a 7-point Likert scale (1 = *do not identify at all*, 7 = *strongly identify*). Means were computed after appropriate reverse coding of seven items (range: 1–7). Cronbach’s α for both the normative (Arrighi & Hausmann, 2022) and current samples was 0.87.

#### Priming questionnaires

A sex/gender stereotype priming questionnaire (Hausmann et al., 2009, adapted from Halpern & Tan, 2001) was administered to participants in the sex/gender priming (experimental) condition. Participants in the control condition completed a sex/gender neutral version (Hausmann et al., 2009). Both versions contained 12 items (e.g., *“You are going to meet a person whom you have never met before. What is the probability that this person is male or female given that this person… is bad at reading street maps?”*). In the priming condition, participants provided two probability ratings (percentages) estimating the likelihood that the person described was male or female. In the control condition, the same questions were used, but participants rated the likelihood that the person was European or North American. In both version, probabilities were required to sum to 100%, with values of 50/50 indicating equal likelihood. Item 10 (*“[this person] can imagine abstract objects and rotate them mentally in all directions.”*) directly referred to mental rotation.

#### Mental rotation test

Spatial performance was measured using the Revised Vandenberg and Kuse Mental Rotations Tests (version MRT-A; Peters et al., 1995). The test consisted of two sets of 12 items. Each item displayed one target figure and four comparison figures. Two of the comparison figures depicted rotated version of the target (correct matches), while the other two depicted different objects. Participants received one point per item if both correct matches were identified (maximum = 24 points). Participants were given 4 min per set (8 min in total).

#### Mirror pictures task

The Mirror Pictures (MP) subtest of the WILDE Intelligenz-Test (Jäger & Althoff, 1994) was also used. The test comprised of 24 items, each presenting five simple line drawings. Four drawings were rotated versions of the same object, and one was a mirrored version. Participants identified the mirrored figure, earning one point per correct response (maximum = 24 points). The total time limit was 4 min.

#### Self-confidence in MRT and MP

Following Estes & Felker (2012), after each MRT and MP item, participants rated their confidence that their previous response was correct on a 7-point Likert scale (1 = *not at all confident*, 7 = *extremely confident*). Only self-confidence ratings for attempted items were included in the analysis.

### Procedure

All testing took place in a controlled laboratory environment. Participants were tested individually by one of two researchers, both cisgender women.

Prior to cognitive testing, participants completed the self-efficacy and spatial anxiety scales. Participants were randomly assigned, through counterbalancing, to either the sex/gender priming or neutral control condition and corresponding questionnaire. Subsequently, they completed the MRT and MP tasks in counterbalanced order, providing confidence ratings after each trial.

After completing the cognitive task, participants provided demographic information and were fully debriefed. Saliva samples were also collected as part of a broader project but were not analysed for the purpose of this article.

The total testing session lasted approximately 60 min.

## Results

Statistical analyses were conducted using SPSS v29. Raw data and code are available at https://osf.io/3bcag/overview?view_only=423b3d06a7104c5https://osf.io/tvdpu/overview?view_only=d54cf334a2d2421999ea763832a9c81e.

### Mental rotation performance

**Fig. 1:**
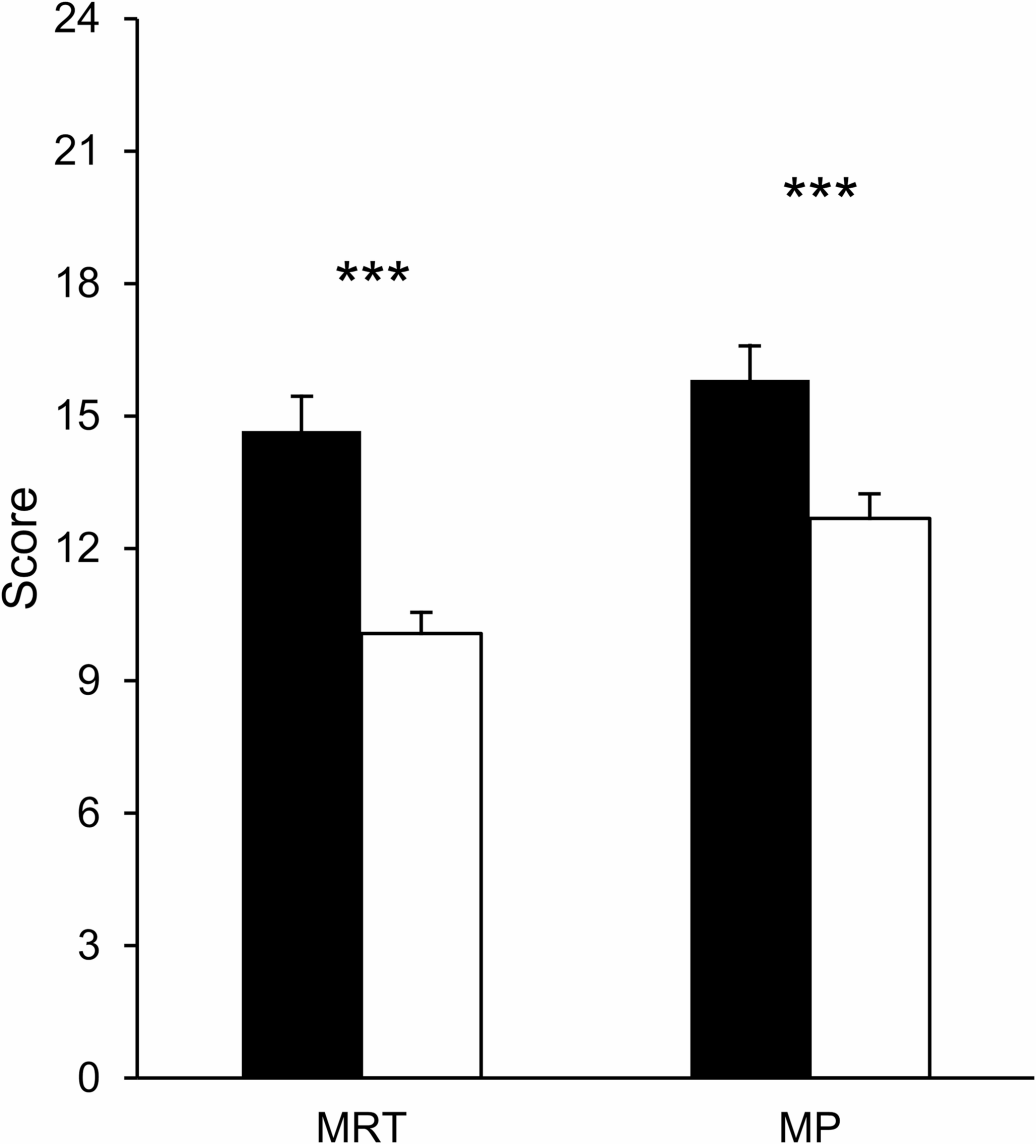
Mean (and SEMs) MRT and MP scores. Mean mental rotations test (MRT) and mirror pictures (MP) test scores (error bars indicate SEMs). Black bars represent males, and white bars represent females. Sex/gender differences favouring males were significant for both MRT and MP (***p <.001)

Two 2 × 2 ANOVAs with sex/gender (male, female) and condition (sex/gender primed, control) as between-subject factors were conducted on MRT and MP scores to test the effects of sex/gender priming on males’ and females’ mental rotation performance (Hypothesis 1; see Fig. 1). For the MRT, the main effect of sex/gender was significant, *F*_(1, 126)_ = 27.70, *p* <.001, *η*_*p*_^*2*^ = 0.18, with males, 14.66 ± 5.58, scoring higher than females, 10.07 ± 4.31. No other main effects or interactions approached significance, all *F* < 2.06, *p* >.153.

For the MP task, the main effect of sex/gender was also significant, *F*_(1, 126)_ = 11.47, *p* <.001, *η*_*p*_^*2*^ = 0.08, with males, 15.82 ± 5.49, outperforming females, 12.68 ± 4.93. No other main effects or interactions approached significance, all *F* < 1.49, *p* >.225, indicating no influence of sex/gender priming on cognitive performance (hence, Hypothesis 3 was not tested).

Because a considerable proportion of male participants had a STEM background, an exploratory analysis examined whether field of study (coded as STEM (including biology) versus non-STEM) accounted for the relatively large sex/gender effects. Previous studies suggest that STEM experience enhances mental rotation performance (e.g., Hausmann, 2014; Moè et al., 2021).

For the MRT, the main effect of sex/gender remained significant but was smaller, *F*_(1, 125)_ = 11.68, *p* <.001, *η*_*p*_^*2*^ = 0.09, even after controlling for field, which itself showed a significant effect, *F*_(1, 125)_ = 19.64, *p* <.001, *η*_*p*_^*2*^ = 0.14. No other main effects or interactions approached significance, all *F* < 1.21, *p* >.274.

For the MP task, the effect of field was significant, *F*_(1, 125)_ = 30.37, *p* <.001, *η*_*p*_^*2*^ = 0.20, whereas the main effect of sex/gender was no longer significant, *F*_(1, 125)_ = 1.61, *p* =.208, *η*_*p*_^*2*^ = 0.01. This suggests that the apparent sex/gender difference in this easier task was largely attributable to the overrepresentation of males with STEM background. No other main effects or interactions approached significance, all *F* < 0.65, *p* >.421.

### Self-perception

#### Self-confidence

Two 2 × 2 ANOVAs with sex/gender and condition as between-subject factors were conducted on MRT and MP self-confidence scores (Hypothesis 2).

For MRT self-confidence, there was a significant main effect of sex/gender, *F*_(1, 126)_ = 20.70, *p* <.001, *η*_*p*_^*2*^ = 0.14, with males, 5.64 ± 0.96, reporting higher self-confidence than females, 4.67 ± 1.29. No other main effects or interactions approached significance, all *F* < 0.316, *p* >.574.

For MP self-confidence, the main effect of sex/gender was again significant, *F*_(1, 126)_ = 9.88, *p* =.002, *η*_*p*_^*2*^ = 0.07, with males, 6.15 ± 1.01, reporting higher self-confidence than females, 5.51 ± 1.18. No other main effects or interactions approached significance, all *F* < 0.181, *p* >.671.

The same exploratory analyses with field of study as a covariate were carried out. For the MRT, self-confidence, the main effect of sex/gender remained significant but was smaller, *F*_(1, 125)_ = 11.41, *p* <.001, *η*_*p*_^*2*^ = 0.08, even after controlling for field, which itself showed a significant effect, *F*_(1, 125)_ = 4.70, *p* =.032, *η*_*p*_^*2*^ = 0.04. No other main effects or interactions approached significance, all *F* < 0.47, *p* >.498.

For the MP test, self-confidence, the main effect of sex/gender remained significant but was smaller, *F*_(1, 125)_ = 5.71, *p* =.018, *η*_*p*_^*2*^ = 0.04. No other main effects or interactions approached significance, all *F* < 1.61, *p* >.206.

#### Spatial anxiety

A 2 × 2 ANOVA revealed a significant main effect of sex/gender on baseline spatial anxiety, *F*_(3, 126)_ = 17.37, *p* <.001, *η*_*p*_^*2*^ = 0.12, with males, 3.08 ± 0.98, reporting lower spatial anxiety than females, 3.79 ± 0.92. No other main effects or interactions approached significance, all *F* < 1.33, *p* >.251.

The same exploratory analyses with field of study as a covariate were carried out. The main effect of sex/gender remained significant but was smaller, *F*_(1, 125)_ = 5.07, *p* =.026, *η*_*p*_^*2*^ = 0.04, even after controlling for field, which itself showed a significant effect, *F*_(1, 125)_ = 22.74, *p* <.001, *η*_*p*_^*2*^ = 0.15. No other main effects or interactions approached significance, all *F* < 0.97, *p* >.326.

#### Self-efficacy

A 2 × 2 ANOVA revealed a significant main effect of sex/gender on baseline self-efficacy, *F*_(3, 126)_ = 18.20, *p* <.001, *η*_*p*_^*2*^ = 0.13, with males, 4.78 ± 0.84, reporting higher self-efficacy than females, 4.07 ± 0.97. No other main effects or interactions approached significance, all *F* < 0.91, *p* >.341.

The same exploratory analyses with field of study as a covariate were carried out. The main effect of sex/gender remained significant but was smaller, *F*_(1, 125)_ = 12.21, *p* <.001, *η*_*p*_^*2*^ = 0.09. No other main effects or interactions approached significance, all *F* < 1.15, *p* >.286.

### Endorsement of sex/gender stereotypes

Exploratory analyses examined sex/gender stereotypes endorsed by participants in the experimental condition only (*n* = 65; 25 males, 40 females) before cognitive testing. The probability estimates of the person being male were used as the dependent measure, where values above 50% indicated a bias toward *male*, and values below 50% indicated a bias toward *female*.

To assess whether participants endorsed sex/gender stereotypes, Bonferroni-corrected (here and below, the correction was applied to the significance level rather than modifying *p* values) one-sample *t*-tests (test value = 50%) were conducted separately for males and females. Female participants, 54.63% ± 7.88, and, marginally, male participants, 53.00% ± 5.00, rated the person described in Item 10 (*…can imagine abstract objects and rotate them mentally in all directions*) as more likely to be male, *t*_(39)_ = 3.71, *p* <.001, *d* = 0.59, and *t*_(24)_ = 3.00, *p* =.006, *d* = 0.60, respectively. Male probability estimates for Item 6 (*…is bad at reading street maps*) also differed significantly from 50%, *t*_(24)_ = −3.73, *p* =.001, *d* = −0.75. For females, additional items significantly differed from 50% (*p* <.004; see Table 2). No other items showed significant deviations, all *t* < 4.75, *p* >.010.

**Table 2:** Gender stereotype questionnaire items and mean probability estimates for participants of the experimental condition

Item	Probability estimates of being male				
	**M**		**F**		
*“You are going to meet a person whom you have never met before. What is the probability that this person is male or female given that this person…”*					
1. …has problems recognizing a complicated drawing when he/she sees it upside-down	47.8		44.3*		
2. …can imagine common objects from different perspectives	51.2		51.0		
3. …can easily remember the names of guests at a party	45.0		44.5		
4. …often makes spelling mistakes	48.8		48.0		
5. …can draw a map of the area where he/she lives	50.8		56.6*		
6. …is bad at reading street maps	44.0*		45.3		
7. …has problems to summarize a book or movie in a short and clear manner	47.8		49.3		
8. …does not use landmarks for orientation	48.6		46.8		
9. …can speak three different languages fluently	59.0		63.6*		
10. …can imagine abstract objects and rotate them mentally in all directions	53.0		54.6*		
11. …often forgets where common objects like keys were put	49.8		47.9		
12. …can generate many words beginning with the same letter in one minute	50.6		53.1		

Results are shown separately for male (M) and female (F) participants of the experimental condition.

Bold probability estimates indicate significant differences from probability estimates of 50%, indicating significant endorsement of sex/gender stereotypes (* *p* <.004).

Independent *t*-tests (Bonferroni-corrected) comparing males and females on each item revealed no sex/gender differences in stereotype endorsement, all *t*_(63)_ < −2.10, *p* >.039.

### Predictors of mental rotation performance

Exploratory simultaneous multiple linear regressions were conducted separately for males and females to identify predictors of mental rotation performance (see Table 3). MRT or MP scores served as dependent variables, with spatial anxiety and task-specific self-confidence as predictors. In additional models only including participants of the experimental condition, endorsement of sex/gender stereotypes about mental rotation (Item 10 probability ratings) was added as predictor. All predictors were *z*-standardised prior to analysis.

**Table 3:** Multiple linear regressions (unstandardised *B* coefficients) for predictors of mental rotation performance

Sample	Task	Sex/gender	SA	SC	G St	*R*^2^	*p*	*n*
Total	MRT^a^	M	−0.46	3.89**	-	0.32	< .001	50
		F	−0.61	1.91***	-	0.26	< .001	80
	MP^a^	M	−1.58	0.83	-	0.11	.025	50
		F	−1.47*	1.40**	-	0.18	< .001	80
Sex/gender primed	MRT^b^	M	−1.74	2.50^+^	−1.98	0.32	.011	25
		F	−0.45	2.21***	−1.97**	0.37	< .001	40
	MP^b^	M	−1.05	2.01	−0.49	0.04	*ns*	25
		F	−2.06*	1.47*	−1.27	0.22	.008	40

Results are shown separately for male (M) and female (F) participants. *Z*-scored predictors: spatial anxiety (SA); self-confidence in the MRT or in the MP (SC); endorsement of sex/gender stereotypes related to mental rotation (G St). Unstandardised *B* coefficients represent the expected change in MRT or MP score for a one-SD increase in each predictor. Coefficients of determination (adjusted *R*^2^) and significances (*p*-level) indicate the proportion of variance explained by the predictors.

^a^ All participants; ^b^ Participants of the sex/gender priming condition only.

Bold unstandardised *B* coefficients are significant.

^+^*p* <.10, *p <.05, **p <.01, ***p <.001, *ns* (not significant), - (not available).

In the overall sample, self-confidence significantly predicted MRT performance for both males, *β* = 0.53, *p* =.001, *F*_(2, 47)_ = 12.32, *p* <.001, adj. *R*^2^ = 0.32, and females, *β* = 0.45, *p* <.001, *F*_(2, 77)_ = 14.11, *p* <.001, adj. *R*^2^ = 0.26. For MP, the regression model was significant for males, *F*_(2, 47)_ = 3.99, *p* =.025, adj. *R*^2^ = 0.11, although no individual predictors were significant. For females, both spatial anxiety, *β* = − 0.27, *p* =.012, and self-confidence, *β* = 0.29, *p* =.007, significantly predicted MP scores, *F*_(2, 77)_ = 9.44, *p* <.001, adj. *R*^2^ = 0.18.

The regressions were repeated only among participants of the sex/gender priming condition and with the addition of endorsement of sex/gender stereotypes about mental rotation as a predictor. Self-confidence was a marginal predictor, *β* = 0.36, *p* =.091, of males’ MRT performance, *F*_(3, 21)_ = 4.81, *p* =.011, adj. *R*^2^ = 0.32. For females, self-confidence was a significant predictor, *β* = 0.50, *p* <.001, and endorsement of sex/gender stereotypes about mental rotation also emerged as a significant predictor, *β* = − 0.38, *p* =.005, *F*_(3, 36)_ = 8.59, *p* <.001, adj. *R*^2^ = 0.37, overall explaining 37% of the variance in females' mental rotation performance. Importantly, the stronger females’ endorsement of the stereotype that “*men are better at mental rotation*,” the lower their MRT performance.

For MP, while the regression model was not significant in males of the sex/gender priming condition, both spatial anxiety, *β* = − 0.36, *p* =.016, and self-confidence, *β* = 0.30, *p* =.046, significantly predicted MP performance in females, *F*_(3, 36)_ = 4.63, *p* =.008, adj. *R*^2^ = 0.22.

## Discussion

The present study investigated the mechanisms underlying cognitive sex/gender differences using a psychosocial approach. Consistent with Hypothesis 1, the well-known male advantage in mental rotation was replicated (Zell et al., 2015), with large and medium effect sizes for the MRT (*d* = 0.95) and MP task (*d* = 0.61), respectively. The effect sizes fall within the upper range of those reported in previous meta-analyses (Hyde, 2016; Reilly & Neumann, 2013; Voyer et al., 1995) and exceed those found in studies using the same tasks (Arrighi & Hausmann, 2022; Hausmann et al., 2009). This discrepancy likely reflects the academic and professional composition of the current sample, which included a higher proportion of males (50%) than females (13.8%) with a STEM background. Given that STEM experience has been associated with increased mental rotation performance (Hausmann, 2014; Moè et al., 2021), this imbalance may have favoured males, contributing to the relatively larger sex/gender differences observed in this study. Indeed, when controlling for participants’ STEM background, sex/gender differences in performance either remained significant but smaller (MRT) or were no longer significant (MP).

As predicted (Hypothesis 2), significant sex/gender differences emerged in self-beliefs. Males reported lower spatial anxiety (*d* = −0.75), higher self-efficacy (*d* = 0.77), and greater self-confidence than females. These results replicate a previously observed robust pattern (Alvarez-Vargas et al., 2020; Arrighi & Hausmann, 2022; Cooke-Simpson & Voyer, 2007; Estes & Felker, 2012; Lyons et al., 2018; Matthews et al., 2024; Towle et al., 2005). Sex/gender differences in self-beliefs became smaller but remained significant when controlling for participants’ STEM background. Notably, sex/gender differences in self-confidence were smaller for the MP (*d* = 0.57) than for the MRT (*d* = 0.83), further supporting the interpretation—consistent with previous findings (Arrighi & Hausmann, 2022)—that participants perceived the MRT as relatively more demanding.

A primary aim of this study was to investigate the effect of sex/gender priming on self-confidence and cognitive performance. Contrary to our prediction (Hypothesis 1), sex/gender priming did not significantly influence participants’ self-confidence or (spatial) cognitive performance, regardless of sex/gender. This finding was surprising given prior evidence of *stereotype threat* effects on women’s mental rotation performance (e.g., Guizzo et al., 2019; Moè, 2009; Moè & Pazzaglia, 2006; Wraga et al., 2006) and *stereotype boost* effects on men’s performance (e.g., Guizzo et al., 2019; Hausmann et al., 2009). One explanation for the absence of priming effects relates to participants’ academic backgrounds and ethnicity. Most female participants were psychology students, and a substantial proportion were Asian—groups that may have been less susceptible to the intended stereotype-related pressure.

Consistent with this reasoning, Hausmann (2014) found that *stereotype threat* effects on mental rotation performance emerged only among women in arts disciplines (i.e., English, Philosophy), suggesting that women in non-STEM, non-psychology fields may be more susceptible to *stereotype threat* in traditionally male-dominated cognitive domains such as spatial abilities. Accordingly, the academic discipline of participants may substantially influence the likelihood of observing sex/gender differences and stereotype effects. Additionally, specifically regarding ethnicity, Asian females within our sample may not have interpreted the sex/gender priming manipulation and/or the mental rotation tasks as threatening, partly due to the widespread stereotype that Asian people excel at mathematical and visuospatial tasks—this stereotype may have served a protective function for this sub-group of participants. In support of this explanation, a previous study has shown that activating Asian-American women’s ethnic identity boosted math performance (Shih et al., 1999). For male participants, the absence of priming effects may reflect a ceiling effect, as MRT performance was already higher than that reported for sex/gender primed males in previous studies (e.g., Hausmann et al., 2009; Hirnstein et al., 2014).

Another explanation for the lack of sex/gender priming effects is the relatively low endorsement of sex/gender stereotypes in the present sample compared to earlier studies using similar paradigms (Hausmann et al., 2009; Hirnstein et al., 2014). This may be attributable to the participants’ younger age, as younger cohorts tend to express weaker endorsement of sex/gender stereotypes (Bhatia & Bhatia, 2021; deMayo et al., 2021; Plante et al., 2019; Wood et al., 2022), and/or to social desirability bias. Probability estimates for Item 10 (reflecting beliefs about mental rotation ability) were significantly different from 50% for both sexes/genders but were smaller in magnitude (53.0–54.6%) than in previous studies (60.4–68.2%; Hausmann et al., 2009; Hirnstein et al., 2014). This pattern aligns with recent findings of reduced sex/gender stereotype endorsement among young adults (Bhatia & Bhatia, 2021; deMayo et al., 2021; Plante et al., 2019; Wood et al., 2022) but could also reflect underreporting due to social desirability. Explicit self-report measures, such as those used here, may underestimate implicit stereotypes that could be more accurately captured by implicit measures like the Implicit Association Test (Greenwald et al., 1998; Greenwald & Banaji, 1995).

Further support for the importance of stereotype endorsement comes from the regression findings. Although sex/gender priming did not affect overall performance, females’ probability estimates for Item 10 (i.e., their endorsement of traditional sex/gender stereotypes regarding mental rotation) negatively predicted their MRT performance. This relationship was observed only in the more demanding MRT, consistent with prior evidence that *stereotype threat* effects are more pronounced under high task demands (e.g., Allison et al., 2017; Keller, 2007) and that stereotype endorsement predicts poorer performance specifically on difficult spatial tasks (Hausmann et al., 2009). The present data thus suggest that sex/gender stereotype endorsement can lead to lower performance among women, particularly when cognitive demands are high.

The regression analyses also revealed that beliefs in one’s spatial abilities were consistent predictors of (spatial) cognitive performance, particularly among females. This contrasts with previous findings in which self-confidence and spatial anxiety were correlated with mental rotation performance for both sexes/genders (Arrighi & Hausmann, 2022) and may be related to the lower size of the male compared to the female sample in the current study. Another likely reason is that males in the current sample showed more positive and less variable self-perceptions of their spatial abilities compared to previous similar studies, possibly inflated by the high proportion with STEM backgrounds. This pattern is consistent with previous research showing that, in certain cases, men’s self-assessment of their spatial abilities can be higher than that of women’s even in the absence of objective sex/gender differences in performance (Ariel et al., 2018; Bordalo et al., 2019; Ehrlinger & Dunning, 2003).

Overall, self-confidence positively predicted performance, whereas spatial anxiety was a negative predictor. Critically, spatial anxiety was linked to performance only in the less demanding MP task. The absence of an effect for the MRT contradicts previous findings (Alvarez-Vargas et al., 2020; Arrighi & Hausmann, 2022) but aligns with Desme et al. (2024), who found that self-confidence—but not spatial anxiety—predicted MRT performance. Methodological differences likely account for inconsistencies across studies, such as the timing of anxiety measurements (before versus after testing). In the present study, self-confidence was measured trial-by-trial, closely aligning with performance and task engagement, whereas spatial anxiety was measured prior to cognitive testing. This difference may have contributed to the higher task-specificity of the self-confidence measure, which could explain why it was a more consistent predictor of performance.

Moreover, females’ greater effort in the MRT compared to the MP may explain why spatial anxiety predicted performance only in the MP. This aligns with evidence that increased effort can buffer anxiety-related performance declines (Eysenck, 2010; Eysenck et al., 2007; Eysenck & Calvo, 1992; Vytal et al., 2012).

Another possible explanation for the lack of association between spatial anxiety and MRT performance is that measuring spatial anxiety before cognitive testing could underestimate females’ spatial anxiety, weakening the relationship with performance. However, spatial anxiety is considered a stable self-concept variable in adulthood (Muffato et al., 2023), suggesting that early measurement would not necessarily underestimate women’s anxiety levels. Additionally, while recent evidence indicates that women may lower their self-perception of spatial abilities after completing the MRT (Arrighi et al., 2026), this likely reflects a later overestimation of low self-beliefs rather than an initial underestimation, consistent with previous reports of women’s tendency to underestimate their spatial abilities (Hofer et al., 2025).

While self-confidence was a consistent predictor of mental rotation performance, an important unresolved question concerns the existence and direction of a causal relationship between self-confidence and performance. A recent report by Arrighi et al. (2026) provides some support for such a link, showing that the positive effect of false positive feedback on mental rotation performance was mediated by an increase in self-confidence. However, because confidence was measured after each trial, these ratings may themselves have been influenced by task performance, raising the possibility of reverse causality. Consequently, the direction of the causal pathway remains unclear: self-confidence may influence performance, performance may influence self-confidence, or the relationship may be reciprocal.

Evidence from the mathematical cognition literature suggests that such relationships are often bidirectional. A recent longitudinal meta-analysis by Pelegrina et al. (2025) reported reciprocal associations of similar magnitude between math anxiety and math performance. A similar bidirectional relationship may therefore exist between confidence and performance in mental rotation. Future research should aim to (1) clarify potential order effects by comparing confidence-performance relationships when confidence is assessed before versus after each trial, and (2) examine these relationships using longitudinal designs to better understand the direction of causality.

Several limitations of the present study warrant consideration. Although the sample size was at the upper end of those used in comparable studies (Cooke-Simpson & Voyer, 2007; Guizzo et al., 2019; Hausmann et al., 2009; Hirnstein et al., 2009, 2014; *n* = 34–149) and met the power analysis requirements, recruitment was somewhat biased toward psychology and STEM participants. However, including field (STEM versus non-STEM) as a covariate did not substantially alter the results, and the relatively large sample size supports the robustness and generalisability of the findings.

Future research could examine whether interventions that reduce sex/gender stereotype endorsement lead to improved (spatial) cognitive performance, particularly among women (e.g., Hausmann et al., 2009). Additionally, given evidence that effort may buffer anxiety’s impact on (spatial) performance, future research should explicitly test effort as a moderating variable (e.g., Eysenck, 2010). Finally, although biological factors such as stress and sex hormones were not central to this study, they may contribute to the mechanisms linking stereotypes and self-perception to performance, potentially through a physiological stress response. Future research should consider integrating biological (e.g., hormone levels), psychological (e.g., self-beliefs), and sociocultural (e.g., stereotype endorsement) factors within a single paradigm. An effective procedure may be to measure salivary hormones and/or physiological stress indicators (e.g., heart rate, blood pressure) before, during, and after cognitive testing (e.g., Hausmann et al., 2009; Sanchis-Segura et al., 2018).

### Conclusions

The present study replicated previously observed sex/gender differences in both beliefs about one’s (spatial) abilities and cognitive performance, consistent in both magnitude and direction with previous literature. Although sex/gender priming did not significantly influence self-confidence or cognitive performance, beliefs about one’s cognitive abilities and endorsement of sex/gender stereotypes emerged as consistent predictors of performance in sex-/gender-sensitive tasks, particularly among females. These results suggest that sex/gender differences in (spatial) cognitive tasks (i.e., men outperforming women), primarily emerge when women hold lower beliefs in their spatial abilities and stronger traditional sex/gender stereotypes. The findings underscore the importance of routinely assessing both self-beliefs and stereotype endorsement when investigating cognitive group differences, and of considering their independent and interactive effects with task demands.

## Data Availability

The full dataset and code are available on the Open Science Framework (https://osf.io/3bcag/).

## Footnotes

1 The terms sex and gender are often used interchangeably in academic literature, although they refer to different concepts. Birth-assigned sex relates to an individual’s anatomical and physiological characteristics, while gender refers to someone’s gender identity and expression formed in relation to societal ideas about gender. In line with recent suggestions (e.g., Hausmann, 2021), we used the term *sex/gender* throughout, since it is not always clear whether some of these individual differences are related to sex and/or gender. Additionally, we used the terms *man* and *woman* for ease, unless referring to the sample of the current study, for which we specified both sex and gender characteristics.
